# Supplementary material for: Maternal Sleep Problems in the Periconceptional Period and the Impact on Health of Mother and Offspring: A Systematic Review
Source: Clocks Sleep. 2022 Nov 24;4(4):688–713. doi: 10.3390/clockssleep4040052 (PMC9777334; doi:10.3390/clockssleep4040052)
Supplement: Supplementary file 1 [file clockssleep-04-00052-s001.zip › clockssleep-1916074-supplementary.pdf]

Appendix. Search terms used in the current systematic review

Embase.com
(sleep/de OR 'night sleep'/exp OR 'sleep debt'/exp OR 'sleep pattern'/exp OR 'sleep quality'/exp OR 'sleep stage'/exp OR 'sleep time'/exp OR 'sleep waking cycle'/exp OR 'sleep deprivation'/de OR 'sleep parameters'/exp OR 'sleep disorder'/de OR 'circadian rhythm sleep disorder'/exp OR 'fragmented sleep'/exp OR 'hypersomnia'/exp OR 'insomnia'/exp OR 'parasomnia'/exp OR 'sleep disordered breathing'/exp OR (sleep* OR insomnia* OR hypersomn* OR hyposomn* OR dyssomni* OR parasomni* OR (circadian* NEAR/3 disrupt*)):ab,ti) AND (pregnancy/exp OR 'pregnant woman'/de OR 'prepregnancy care'/de OR conception/de OR 'pregnancy outcome'/exp OR 'pregnancy complication'/mj OR 'pregnancy disorder'/mj OR 'spontaneous abortion'/de OR (pregnan* OR prepregnancy OR preconception* OR periconception* OR pre-conception* OR peri-conception* OR (concepti* NEAR/3 (before OR around OR period*)) OR (spontan* NEAR/3 abortion*) OR miscarriage*):ab,ti) NOT ([animals]/lim NOT [humans]/lim) NOT [conference abstract]/lim AND [english]/lim NOT ('case report'/de OR case-report:ti)

Medline ALL Ovid
(Sleep/ OR Sleep Hygiene/ OR exp Sleep Stages/ OR Sleep Deprivation/ OR Sleep Wake Disorders/ OR Sleep Initiation and Maintenance Disorders/ OR Sleep Disorders, Circadian Rhythm/ OR Disorders of Excessive Somnolence/ OR exp Parasomnias/ OR Sleep Apnea Syndromes/ OR (sleep* OR insomnia* OR hypersomn* OR hyposomn* OR dyssomni* OR parasomni* OR (circadian* ADJ3 disrupt*)).ab,ti.) AND (exp Pregnancy/ OR Pregnant Women/ OR Preconception Care/ OR Fertilization/ OR exp Pregnancy Outcome/ OR * Pregnancy Complications/ OR Abortion, Spontaneous/ OR (pregnan* OR prepregnancy OR preconception* OR periconception* OR pre-conception* OR peri-conception* OR (concepti* ADJ3 (before OR around OR period*)) OR (spontan* ADJ3 abortion*) OR miscarriage*).ab,ti.) NOT (exp animals/ NOT humans/) AND english.la. NOT (case reports/ OR case-report.ti.)

Web of Science
TS=(((sleep* OR insomnia* OR hypersomn* OR hyposomn* OR dyssomni* OR parasomni* OR (circadian* NEAR/2 disrupt*))) AND ((pregnan* OR prepregnancy OR preconception* OR periconception* OR pre-conception* OR peri-conception* OR (concepti* NEAR/2 (before OR around OR period*)) OR (spontan* NEAR/2 abortion*) OR miscarriage*))) AND DT=(article) AND LA=(english) NOT TI=("case report")

Cochrane CENTRAL
((sleep* OR insomnia* OR hypersomn* OR hyposomn* OR dyssomni* OR parasomni* OR (circadian* NEAR/3 disrupt*)):ab,ti) AND ((pregnan* OR prepregnancy OR preconception* OR periconception* OR pre NEXT conception* OR peri NEXT conception* OR (concepti* NEAR/3 (before OR around OR period*)) OR (spontan* NEAR/3 abortion*) OR miscarriage*):ab,ti)

Table S1. Extensive overview of associations of included studies regarding maternal sleep problems in the periconceptional period (N=27).

Study	Outcomes
Bublitz *et al.* (2010)	Both cross-sectionally (F=0.74; p=.39) and longitudinally (F=.43; p=.51), no statistic significant association was found between self-reported measures of sleep disordered breathing at 12 weeks of gestation, as measured by the Berlin Questionnaire.<br><br>Cross-sectionally, the authors did not find a statistical significant association between objective measures of sleep disordered breathing at 12 weeks of gestation, as measured by the apnea-hypopnea index (AHI).<br>Longitudinally, associations were found in late pregnancy (32 weeks of gestation). This was found unadjusted (β=0.20; SE=1.89; p=.026), adjusted for various covariates (β=0.22; SE=1.89; p=.012), when the sleep item was additonally removed from the Patient Health Questionnaire-9 (β=0.25; SE=1.60; p=0.004), and when women who used antidepressants were removed from the analyses (β=0.19; SE=1.99; p=0.043).
Chang *et al.* (2015)	PSQI subscale sleep latency was associated with depression, assessed with the Edinburgh Postnatal Depression Scale (effect size 1.27; p<.05), and with fruit and vegetable intake, assessed with the Rapid Food Screener (effect size 2.17; p<.05) in the first trimester.
Doyon *et al.* (2020)	More hours of self-reported sleep was associated with lower blood glucose at 1 hour post 50 g (β=0.013 of log-transformed glucose for each hour of sleep; SE=0.007; p=.06). The strength of the association was reduced after adjusting for confounders (β=0.009; SE=0.007; p=.17).<br>Models estimated 1 hour post 50 g glucose values at 5.60 mmol/L (95%CI 5.35-5.86) for women sleeping 5 hours per night, 5.43 (95%CI 5.34-5.52) for 8 hours per night and 5.26 (95%CI 5.06-5.46) for 11 hours of sleep per night.
Facco *et al.* (2018)[a]	Self-reported short sleep duration (<7 hours) in the first trimester was associated with hypertensive disorders (OR 1.31; 95%CI 1.10-1.55; p=.002) and gestational diabetes (OR 1.45; 95%CI 1.10-1.92; p=.009). Results were not significant after adjustment for confounders: hypertension aOR 1.19 (95% CI 1.00-1.42; p=.053); gestational diabetes aOR 1.23 (95%CI 0.92-1.64; p=.164).<br><br>Late sleep midpoint (after 5 AM) in the first trimester was not associated with hypertensive disorders (OR 1.22; 95%CI 1.00-1.49; p=.055), which remained after adjusting for confounders (aOR 1.15; 95%CI 0.92-1.43; p=.216).<br>Late sleep midpoint (after 5 AM) in the first trimester was not associated with gestational diabetes (OR 1.31; 95%CI 0.94-1.82; p=.111). When confounded for only age and BMI, the association was statistically significant (aOR 1.67; 95%CI 1.17-2.38; p=.004). However, this disappeared after adjustment for further confounders (aOR 1.37; 95%CI 0.95-1.98; p=.089)
Facco *et al.* (2019)[a]	Self-reported short sleep duration (<7 hours) in the first trimester was not associated with preterm birth (crude: OR 1.17; 95%CI 0.94-1.47; p=.16; adjusted: aOR 1.15 (95%CI 0.92-1.44; p=.23) and spontaneous preterm birth (crude: OR 1.11; 95%CI 0.84-1.46; p=.48; adjusted: aOR 1.11; 95%CI 0.84-1.47; p=.47).

	Late sleep midpoint (after 5 AM) in the first trimester was associated with a higher rate of preterm birth (9.5% vs 6.9%; p=.005) and of spontaneous preterm birth (6.2% vs 4.4%; p=.019), compared to women with an early sleep midpoint (before 5 AM).<br>Crude OR for preterm birth 1.42 (95%CI 1.11-1.82; p=.005) and spontaneous preterm birth 1.43 (95%CI 1.06-1.93; p=.019).<br>After adjustment for age and BMI, the association remained with preterm birth (aOR 1.39; 95%CI 1.08-1.80, p=.01) and spontaneous preterm birth (aOR 1.45; 95%CI 1.06-1.99, p=.02). After further adjustment, the association remained for preterm birth (aOR 1.34; 95%CI 1.03-1.74; p=.03), but not for spontaneous preterm birth (aOR 1.34; 95%CI 0.97-1.85; p=.07).
Franco-Sena *et al*. (2018)	Among nulliparous women, 24-h sleep duration in the first trimester was inversely associated with infant's body weight z-score (crude; β=-0.35; 95%CI 0.57-0.14; p=.07; for model 1: β=0.44; 95%CI 0.68-0.21; p<.001; for model 2: β=-0.42; 95%CI 0.65-0.18; p<.001).<br>First trimester nightly sleep duration was associated with infant's body weight z-score (β=-0.28; 95%CI 0.53-0.03; p=.029), but not after adjusting for confounders (β=-0.27; 95%CI 0.58-0.05; p=.092).<br>First trimester napping sleep duration was not associated with infant's body weight (crude β=-0.08; 95%CI 0.60-0.44; p=.757; adjusted β=-0.20; 95%CI 0.77-0.37; p=.485).<br><br>Among multiparous women, first trimester nightly sleep duration was not associated with infant body weight (crude β=-0.07; 95%CI 0.28-0.13; p=.478; adjusted β=-0.01; 95%CI 0.24-0.22; p=.954).<br>First trimester napping sleep duration was not associated with infant body weight (crude β=-0.13; 95%CI 0.62-0.35; p=.580; adjusted β=0.11; 95%CI 0.49-0.71; p=.719).<br>First trimester 24-h sleep duration was not associated with infant body weight (crude β=-0.09; 95%CI 0.25-0.07; p=.277; adjusted β=-0.36; 95%CI 1.39-0.66; p=.483).
Gelaye *et al*. (2015)	A PSQI score of >5 in the first trimester was associated with suicidal ideation (OR 2.72; 95%CI 1.78–4.16), compared to a PSQI score of ≤5. After adjustment for confounders, the association remained (aOR 2.19; 95% CI 1.40–3.42). After further adjustment for maternal depression, PSQI >5 was still associated with suicidal ideation (aOR 1.67; 95% CI 1.02–2.71).<br><br>When assessed as continuous variable, first trimester total PSQI score was also associated with increased odds for suicidal ideation (crude: OR 1.26; 95%CI 1.17-1.36; model 1: aOR 1.22; 95%CI 1.13-1.32; model 2: aOR 1.18; 95%CI 1.08-1.28).
Georgiou *et al*. (2019)	Women with preeclampsia appear to have higher rates of insomnia (OR 5.03; 95%CI 1.41-17.89; p<.05), less sleep quality (OR 4.45; 95%CI 1.53-12.99; p<.05), and more sleepiness (OR 3.27; 95%CI 1.15-9.31; p<.05) before pregnancy, compared to women without pre-eclampsia.
Haney *et al.* (2014)[b]	Diary-assessed sleep latency at 10-12 weeks was correlated with systolic blood pressure at 14-16 weeks after adjusting for covariates (r(132)=0.18, p=.03), but not after correcting for multiple comparisons.

	Neither diary- nor actigraphy-assessed wake after sleep onset and total sleep time were associated with any of the cardiometabolic factors.
Hill *et al.* (2020)	In models adjusted for covariates, first trimester PSQI score was not associated with gestational weight gain adequacy (for inadequate weight gain: OR 1.00 (95%CI 0.88-1.13; p=.99); for excessive weight gain: OR 0.95 (95%CI 0.86-1.06; p=.35).<br>There was also no association with first trimester sleep duration (for inadequate weight gain: OR 1.03 (95%CI 0.73-1.46; p=.85); for excessive weight gain: OR 1.09 (95%CI 0.84-1.42; p=.50).<br><br>First trimester pregnancy sleep duration and quality were not associated with gestational fat gain (respectively, β=0.01; SE=0.17; p=.97; β=0.03; SE=0.07; p=.66).
Liu *et al.* (2019)	First trimester PSQI was correlated with female infant body weight z-score (r=-0.093; p<.05), but not with male infant body weight z-score (r=0.022; p>.05).<br><br>After adjusting for confounders, first trimester PSQI scores were associated with bodyweight z-scores among female newborns (β = −0.029; 95%CI 0.057-0.001; p=.045). This association remained statistically significant after further adjustment (β = −0.032, 95% CI 0.063-0.001, p=.043).<br>These results were not found for male newborns (model 1: β=0.027; 95%CI 0.002-0.057; p=.071; model 2: β=0.026; 95%CI 0.006-0.058; p=.113).<br><br>First trimester PSQI score was not a risk factor for small for gestational age (model 1: aOR 1.052; 95%CI 0.892-1.241; p=.546; model 2: aOR 1.040; 95%CI 0.865-1.250; p=.678) or low birth weight (model 1: aOR 1.010; 95%CI 0.733-1.391; p=.953; model 2: aOR 1.170; 95%CI 0.821-1.669; p=.386).
Lyu *et al*. (2020)	Less maternal sleep duration in the first trimester was observed when their children had either shorter sleep duration (β=0.113; SE=0.012; p<.001) or sleep problems (β=-0.308; SE=0.072; p<.001).<br><br>Maternal short sleep duration (≤8 hours) in the first trimester could predict both short infant sleep duration (<10 hours; crude: OR 1.24; 95%CI 1.12-1.38; p<.01; adjusted: aOR 1.25; 95%CI 1.12-1.39; p<.01). It could also predict infant sleep disturbance (OR 1.28; 95%CI 1.04-1.59; p<.01), but not after adjustment for confounders (aOR 1.13; 95%CI 0.90-1.42; p>.05).
Marinelli *et al.* (2021)	Each hour of sleep increased neonate birth weights by 44.7 grams in women sleeping <7 hours per day before pregnancy (β 44.72 (95%CI 0.28-89.17); p=0.049). Neonate birth weight decreased by 39.2 grams in women sleeping ≥9 hours of sleep per day before pregnancy (β=-39.22 (95%CI -61.46-16.97); p=.001).<br><br>Associations between maternal sleep duration before pregnancy and birth weight were similar after adjusting for additional confounders.

After adjusting for alcohol:

- ≤7 hours: β=45.27 (95%CI 0.86-89.68); p=0.046
- >7 hours and ≤9 hours: β=15.54 (95%CI -9.55-40.64); p=.225
- >9 hours: β=-38.82 (95%CI -61.05-16.58); p=.001

After adjusting for smoking:

- ≤7 hours: β=39.87 (95%CI -4.26-84.01); p=.077
- >7 hours and ≤9 hours: β=14.08 (95%CI -10.85-39.02); p=0.268
- >9 hours: β=-33.17 (95%CI -55.35-10.99); p=.003

After adjusting for maternal education:

- ≤7 hours: β=39.06 (-5.20-83.32); p=.084
- >7 hours and ≤9 hours: β=14.74 (-10.20-39.68); p=0.247
- >9 hours: β=-31.79 (95%CI -54.04-9.54); p=.005

After adjusting for parity:

- ≤7 hours: β=35.73 (95%CI -7.85-79.30); p=.108
- >7 hours and ≤9 hours: β=18.33 (95%CI -6.27-42.94); p=.144
- >9 hours: β=-33.29 (95%CI -55.15-11.44); p=.003

After adjusting for marital status:

- ≤7 hours: β=39.95 (95%CI -4.18-84.09); p=0.076
- >7 hours and ≤9 hours: β=14.12 (95%CI -10.81-39.05); p=.267
- >9 hours: β=-33.33 (95%CI -55.52-11.14); p=.003

After adjusting for working during pregnancy:

- ≤7 hours: β=41.00 (95%CI -3.18-85.19); p=.069
- >7 hours and ≤9 hours: β=12.38 (95%CI -12.73-37.49); p=.334
- >9 hours: β=-35.06 (95%CI -57.45-12.68); p=.002

After adjusting for mother's social class:

- ≤7 hours: β=38.88 (95%CI -5.25-83.02); p=.084
- >7 hours and ≤9 hours: β=14.13 (95%CI -10.78-39.03); p=.266
- >9 hours: β=-32.61 (95%CI -54.82-10.41); p=.004

After adjusting for total physical activity:

- ≤7 hours: β=39.89 (95%CI -4.24-84.03); p=.076
- >7 hours and ≤9 hours: β=14.09 (95%CI -10.84-39.03); p=.268
- >9 hours: β=-33.21 (95%CI -55.39-11.02); p=.003

	After adjusting for anxiety clinical history reported at first trimester:<br>▪ ≤7 hours: β=39.97 (95%CI -4.23-84.17); p=.076<br>▪ >7 hours and ≤9 hours: β=14.02 (95%CI -10.94-38.97); p=.271<br>▪ >9 hours: β=-33.13 (95%CI -55.32-10.93); p=.003<br><br>After adjusting for depression clinical history reported at first trimester:<br>▪ ≤7 hours: β=39.95 (95%CI -4.25-84.15); p=.076<br>▪ >7 hours and ≤9 hours: β=14.10 (95%CI -10.85-39.05); p=.268<br>▪ >9 hours: β=-33.30 (-55.51-11.09); p=.003
Matsuo *et al.* (2021)	Short sleep duration was associated with a higher risk of postpartum depression (7-8 hours reference group):<br>▪ <6 hours: crude OR 2.06 (95%CI 1.64-2.59); adjusted OR 2.08 (95%CI 1.60-2.70)<br>▪ 6-7 hours: crude OR 1.42 (95%CI 1.21-1.66); adjusted OR 1.41 (95%CI 1.18-1.68)<br>▪ 8-9 hours: crude OR 1.16 (95%CI 0.97-1.38); adjusted OR 1.15 (95%CI 0.94-1.42)<br>▪ ≥9 hours: crude OR 1.20 (95%CI 0.88-1.65); adjusted OR 1.19 (95%CI 0.81-1.76)<br>▪ 1 hour increase: crude OR 0.88 (95%CI 0.83-0.94); adjusted OR 0.86 (95%CI 0.80-0.92)<br><br>Among primipara women, the adjusted OR for high EPDS scores was higher only in women sleeping <6 hours. Multipara women sleeping <6 hours and <6-7 hours showed an increased risk of high EPDS scores.<br><br>Primipara women:<br>▪ <6 hours: crude OR 1.50 (95%CI 1.09-2.05); adjusted OR 1.46 (95%CI 1.02-2.11)<br>▪ 6-7 hours: crude OR 1.23 (95%CI 1.02-1.49); adjusted OR 1.24 (95%CI 0.99-1.54)<br>▪ 8-9 hours: crude OR 1.28 (95%CI 1.03-1.59); adjusted OR 1.22 (95%CI 0.94-1.58)<br>▪ ≥9 hours: crude OR 1.40 (95%CI 0.94-2.08); adjusted OR 1.49 (95%CI 0.92-2.40)<br>▪ 1 hour increase: crude OR 0.98 (95%CI 0.91-1.06); adjusted OR 0.96 (95%CI 0.88-1.06)<br><br>Multipara women:<br>▪ <6 hours: crude OR 3.42 (95%CI 2.42-4.82); adjusted OR 3.26 (95%CI 2.21-4.81)<br>▪ 6-7 hours: crude OR 1.76 (95%CI 1.34-2.33); adjusted OR 1.89 (95%CI 1.38-2.58)<br>▪ 8-9 hours: crude OR 1.25 (95%CI 0.93-1.67); adjusted OR 1.11 (95%CI 0.79-1.58)<br>▪ ≥9 hours: crude OR 1.16 (95%CI 0.68-1.99); adjusted OR 0.90 (95%CI 0.46-1.78)<br>▪ 1 hour increase: crude OR 0.78 (95%CI 0.71-0.86); adjusted OR 0.74 (95%CI 0.66-0.83)
Nakahara *et al.* (2020)[c]	Self-reported sleep duration before pregnancy was not associated with preterm birth (7-8 hours reference group):<br>▪ <6 hours: model 1 aRR 1.08 (95%CI 0.95-1.22); model 2 aRR 1.04 (95%CI 0.92-1.17)<br>▪ 6-7 hours: model 1 aRR 1.08 (95%CI 0.99-1.18); model 2 aRR 1.06 (95%CI 0.97-1.15)<br>▪ 8-9 hours: model 1 aRR 0.99 (95%CI 0.91-1.07); model 2 aRR 1.00 (95%CI 0.92-1.09)<br>▪ 9-10 hours: model 1 aRR 0.93 (95%CI 0.83-1.05); model 2 aRR 0.93 (95%CI 0.83-1.04)

- >10 hours: model 1 aRR 1.04 (95%CI 0.89-1.22); model 2 aRR 1.00 (95%CI 0.85-1.16)

Self-reported bedtime before pregnancy was not associated with preterm birth (21:00-24:00 reference group):

- 24:00-03:00: model 1 aRR 1.02 (95%CI 0.95-1.09); model 2 aRR 1.00 (95%CI 0.94-1.08)
- Other: model 1 aRR 0.99 (95%CI 0.81-1.19); model 2 aRR 0.91 (95%CI 0.75-1.11)

Self-reported sleep duration before pregnancy was not associated with infant awakenings (7-8 hours reference group):

- <6 hours: model 1 aRR 1.02 (95%CI 0.91-1.14); model 2 aRR 1.07 (95%CI 0.96-1.20)
- 6-7 hours: model 1 aRR 0.91 (95%CI 0.84-0.98); model 2 aRR 0.95 (95%CI 0.87-1.02)
- 8-9 hours: model 1 aRR 1.04 (95%CI 0.97-1.11); model 2 aRR 1.00 (95%CI 0.93-1.07)
- 9-10 hours: model 1 aRR 1.05 (95%CI 0.96-1.16); model 2 aRR 0.99 (95%CI 0.90-1.09)
- >10 hours: model 1 aRR 1.03 (95%CI 0.90-1.17); model 2 aRR 0.98 (95%CI 0.86-1.13)

Self-reported bed time before pregnancy was not associated with infant awakenings (21:00-24:00 reference group):

- 24:00-03:00: model 1 aRR 0.96 (95%CI 0.90-1.02); model 2 aRR 1.05 (95%CI 0.98-1.12)
- Other: model 1 aRR 1.13 (95%CI 0.96-1.32); model 2 aRR 1.17 (95%CI 1.00-1.37)

Self-reported sleep duration before pregnancy was associated with infant’s tendency to sleep longer during the day than the night (7-8 hours reference group):

- <6 hours: model 1 aRR 1.24 (95%CI 1.17-1.31); model 2 aRR 1.18 (95%CI 1.12-1.25)
- 6-7 hours: model 1 aRR 1.14 (95%CI 1.09-1.18); model 2 aRR 1.10 (95%CI 1.06-1.15)
- 8-9 hours: model 1 aRR 0.93 (95%CI 0.90-0.97); model 2 aRR 0.98 (95%CI 0.95-1.02)
- 9-10 hours: model 1 aRR 0.87 (95%CI 0.82-0.92); model 2 aRR 0.94 (95%CI 0.89-1.00)
- >10 hours: model 1 aRR 0.92 (95%CI 0.85-0.99); model 2 aRR 0.94 (95%CI 0.88-1.02)

Self-reported bed time before pregnancy was associated with infant’s tendency to sleep longer during the day than the night (21:00-24:00 reference group):

- 24:00-03:00: model 1 aRR 1.28 (95%CI 1.24-1.32); model 2 aRR 1.17 (95%CI 1.13-1.20)
- Other: model 1 aRR 1.20 (95%CI 1.11-1.30); model 2 aRR 1.13 (95%CI 1.04-1.22)

Self-reported sleep duration before pregnancy was associated with bad mood of the infant (7-8 hours reference group):

- <6 hours: model 1 aRR 1.33 (95%CI 1.21-1.47); model 2 aRR 1.12 (95%CI 1.01-1.23)
- 6-7 hours: model 1 aRR 1.26 (95%CI 1.17-1.35); model 2 aRR 1.09 (95%CI 1.01-1.16)
- 8-9 hours: model 1 aRR 0.76 (95%CI 0.70-0.82); model 2 aRR 0.97 (95%CI 0.90-1.05)
- 9-10 hours: model 1 aRR 0.68 (95%CI 0.61-0.76); model 2 aRR 1.05 (95%CI 0.94-1.17)
- >10 hours: model 1 aRR 1.00 (95%CI 0.87-1.14); model 2 aRR 1.17 (95%CI 1.02-1.33)

Self-reported bed time before pregnancy was associated with bad mood of the infant (21:00-24:00 reference group):

- 24:00-03:00: model 1 aRR 1.68 (95%CI 1.59-1.77); model 2 aRR 1.12 (95%CI 1.06-1.19)
- Other: model 1 aRR 1.35 (95%CI 1.15-1.57); model 2 aRR 1.10 (95%CI 0.94-1.29)

Self-reported sleep duration before pregnancy was associated with frequent infant crying (7-8 hours reference group):

- <6 hours: model 1 aRR 1.28 (95%CI 1.21-1.35); model 2 aRR 1.17 (95%CI 1.11-1.24)
- 6-7 hours: model 1 aRR 1.18 (95%CI 1.14-1.23); model 2 aRR 1.09 (95%CI 1.05-1.13)

	▪ 8-9 hours: model 1 aRR 0.85 (95%CI 0.82-0.89); model 2 aRR 0.98 (95%CI 0.94-1.02)<br>▪ 9-10 hours: model 1 aRR 0.74 (95%CI 0.69-0.78); model 2 aRR 0.91 (95%CI 0.86-0.97)<br>▪ >10 hours: model 1 aRR 0.94 (95%CI 0.87-1.01); model 2 aRR 1.01 (95%CI 0.94-1.09)<br>Self-reported bed time before pregnancy was associated with frequent infant crying (21:00-24:00 reference group):<br>▪ 24:00-03:00: model 1 aRR 1.38 (95%CI 1.33-1.42); model 2 aRR 1.09 (95%CI 1.06-1.13)<br>▪ Other: model 1 aRR 1.09 (95%CI 1.00-1.20); model 2 aRR 0.96 (95%CI 0.88-1.06)<br><br>Self-reported sleep duration before pregnancy was associated with intense infant crying (7-8 hours reference group):<br>▪ <6 hours: model 1 aRR 1.30 (95%CI 1.23-1.36); model 2 aRR 1.15 (95%CI 1.09-1.20)<br>▪ 6-7 hours: model 1 aRR 1.21 (95%CI 1.17-1.25): model 2 aRR 1.08 (95%CI 1.04-1.12)<br>▪ 8-9 hours: model 1 aRR 0.80 (95%CI 0.77-0.84); model 2 aRR 0.97 (95%CI 0.93-1.01)<br>▪ 9-10 hours: model 1 aRR 0.69 (95%CI 0.65-0.74); model 2 aRR 0.95 (95%CI 0.89-1.00)<br>▪ >10 hours: model 1 aRR 0.88 (95%CI 0.81-0.94); model 2 aRR 0.98 (95%CI 0.91-1.06)<br>Self-reported bed time before pregnancy was associated with intense infant crying (21:00-24:00 reference group):<br>▪ 24:00-03:00: model 1 aRR 1.46 (95%CI 1.42-1.51); model 2 aRR 1.07 (95%CI 1.04-1.10)<br>▪ Other: model 1 aRR 1.14 (95%CI 1.04-1.24); model 2 aRR 0.97 (95%CI 0.89-1.05)
Nakahara *et al.* (2021)[c]	Self-reported sleep duration before pregnancy was not associated with >3 nighttime waking instances (7-8 hours reference group):<br>▪ <6 hours: model 1 aRR 1.04 (95%CI 0.85-1.26); model 2 aRR 1.08 (95%CI 0.89-1.32)<br>▪ 6-7 hours: model 1 aRR 0.95 (95%CI 0.83-1.09); model 2 aRR 0.96 (95%CI 0.84-1.10)<br>▪ 8-9 hours: model 1 aRR 1.10 (95%CI 0.98-1.24); model 2 aRR 1.09 (95%CI 0.96-1.23)<br>▪ 9-10 hours: model 1 aRR 1.23 (95%CI 1.05-1.43); model 2 aRR 1.20 (95%CI 1.02-1.40)<br>▪ >10 hours: model 1 aRR 1.06 (95%CI 0.84-1.35); model 2 aRR 1.07 (95%CI 0.84-1.36)<br>Self-reported bed time before pregnancy was associated with >3 nighttime waking instances (21:00-24:00 reference group):<br>▪ 24:00-03:00: model 1 aRR 0.85 (95%CI 0.76-0.94); model 2 aRR 0.89 (95%CI 0.80-0.99)<br>▪ Other: model 1 aRR 0.83 (95%CI 0.61-1.14); model 2 aRR 0.90 (95%CI 0.65-1.23)<br><br>Self-reported sleep duration before pregnancy was associated with >1 waking instance lasting >1 hour (7-8 hours reference group):<br>▪ <6 hours: model 1 aRR 1.54 (95%CI 1.38-1.71); model 2 aRR 1.49 (95%CI 1.34-1.66)<br>▪ 6-7 hours: model 1 aRR 1.18 (95%CI 1.09-1.28); model 2 aRR 1.16 (95%CI 1.07-1.26)<br>▪ 8-9 hours: model 1 aRR 0.94 (95%CI 0.86-1.02); model 2 aRR 0.96 (95%CI 0.89-1.04)<br>▪ 9-10 hours: model 1 aRR 0.96 (95%CI 0.86-1.07); model 2 aRR 1.00 (95%CI 0.90-1.12)<br>▪ >10 hours: model 1 aRR 1.24 (95%CI 1.08-1.42); model 2 aRR 1.25 (95%CI 1.09-1.44)<br>Self-reported bed time before pregnancy was associated with >1 waking instance lasting >1 hour (21:00-24:00 reference group):<br>▪ 24:00-03:00: model 1 aRR 1.44 (95%CI 1.35-1.53); model 2 aRR 1.38 (95%CI 1.30-1.47)<br>▪ Other: model 1 aRR 1.99 (95%CI 1.73-2.28); model 2 aRR 1.92 (95%CI 1.67-2.21)

Self-reported sleep duration before pregnancy was associated with <8 hour of sleep during the night (20:00-07:59) (7-8 hours reference group):

- <6 hours: model 1 aRR 1.65 (95%CI 1.48-1.84); model 2 aRR 1.60 (95%CI 1.44-1.79)
- 6-7 hours: model 1 aRR 1.21 (95%CI 1.11-1.31); model 2 aRR 1.19 (95%CI 1.09-1.29)
- 8-9 hours: model 1 aRR 0.89 (95%CI 0.82-0.98); model 2 aRR 0.92 (95%CI 0.84-1.00)
- 9-10 hours: model 1 aRR 0.86 (95%CI 0.77-0.97); model 2 aRR 0.90 (95%CI 0.80-1.02)
- >10 hours: model 1 aRR 1.25 (95%CI 1.08-1.44); model 2 aRR 1.26 (95%CI 1.09-1.46)

Self-reported bed time before pregnancy was associated with <8 hour of sleep during the night (20:00-07:59) (21:00-24:00 reference group):

- 24:00-03:00: model 1 aRR 1.37 (95%CI 1.28-1.46); model 2 aRR 1.31 (95%CI 1.22-1.40)
- Other: model 1 aRR 2.11 (95%CI 1.84-2.43); model 2 aRR 2.04 (95%CI 1.77-2.35)

Self-reported sleep duration before pregnancy was associated with falling asleep at 22:00 or later (7-8 hours reference group):

- <6 hours: model 1 aRR 1.39 (95%CI 1.32-1.46); model 2 aRR 1.33 (95%CI 1.26-1.40)
- 6-7 hours: model 1 aRR 1.17 (95%CI 1.13-1.22); model 2 aRR 1.15 (95%CI 1.10-1.19)
- 8-9 hours: model 1 aRR 0.94 (95%CI 0.90-0.97); model 2 aRR 0.96 (95%CI 0.92-1.00)
- 9-10 hours: model 1 aRR 0.80 (95%CI 0.76-0.85); model 2 aRR 0.84 (95%CI 0.79-0.89)
- >10 hours: model 1 aRR 1.02 (95%CI 0.95-1.10); model 2 aRR 1.02 (95%CI 0.95-1.10)

Self-reported bed time before pregnancy was associated with falling asleep at 22:00 or later (21:00-24:00 reference group):

- 24:00-03:00: model 1 aRR 1.60 (95%CI 1.55-1.65); model 2 aRR 1.53 (95%CI 1.48-1.58)
- Other: model 1 aRR 1.40 (95%CI 1.29-1.52); model 2 aRR 1.34 (95%CI 1.23-1.45)

Self-reported sleep duration before pregnancy was not associated with frequency of crying at night (≥5 days/week) (7-8 hours reference group):

- <6 hours: model 1 aRR 1.14 (95%CI 1.03-1.27); model 2 aRR 1.16 (95%CI 1.05-1.29)
- 6-7 hours: model 1 aRR 1.05 (95%CI 0.98-1.13); model 2 aRR 1.05 (95%CI 0.98-1.13)
- 8-9 hours: model 1 aRR 1.05 (95%CI 09.98-1.12); model 2 aRR 1.05 (95%CI 0.98-1.12)
- 9-10 hours: model 1 aRR 1.04 (95%CI 0.95-1.15); model 2 aRR 1.05 (95%CI 0.96-1.15)
- >10 hours: model 1 aRR 0.91 (95%CI 0.79-1.04); model 2 aRR 0.93 (95%CI 0.80-1.07)

Self-reported bed time before pregnancy was not associated with frequency of crying at night (≥5 days/week) (21:00-24:00 reference group):

- 24:00-03:00: model 1 aRR 1.03 (95%CI 0.98-1.09); model 2 aRR 1.05 (95%CI 0.99-1.11)
- Other: model 1 aRR 1.06 (95%CI 0.90-1.24); model 2 aRR 1.11 (95%CI 0.95-1.31)

Self-reported sleep duration before pregnancy was not associated with communication (7-8 hours reference group):

- <6 hours: model 1 aRR 1.19 (95%CI 0.45-3.15); model 2 aRR 1.15 (95%CI 0.43-3.03)
- 6-7 hours: model 1 aRR 1.40 (95%CI 0.75-2.61); model 2 aRR 1.32 (95%CI 0.71-2.47)

- 8-9 hours: model 1 aRR 0.82 (95%CI 0.41-1.63); model 2 aRR 0.90 (95%CI 0.45-1.80)
- 9-10 hours: model 1 aRR 0.95 (95%CI 0.39-2.35); model 2 aRR 1.12 (95%CI 0.45-2.82)
- >10 hours: model 1 aRR 1.29 (95%CI 0.38-4.31); model 2 aRR 1.42 (95%CI 0.42-4.78)

Self-reported bed time before pregnancy was not associated with communication (21:00-24:00 reference group):

- 24:00-03:00: model 1 aRR 1.01 (95%CI 0.59-1.73); model 2 aRR 0.86 (95%CI 0.49-1.50)
- Other: model 1 aRR 1.33 (95%CI 0.32-5.51); model 2 aRR 1.39 (95%CI 0.33-5.79)

Self-reported sleep duration before pregnancy was not associated with gross motor skills (7-8 hours reference group):

- <6 hours: model 1 aRR 1.08 (95%CI 0.95-1.23); model 2 aRR 1.07 (95%CI 0.94-1.22)
- 6-7 hours: model 1 aRR 1.12 (95%CI 1.03-1.22); model 2 aRR 1.11 (95%CI 1.02-1.20)
- 8-9 hours: model 1 aRR 0.99 (95%CI 0.91-1.07); model 2 aRR 1.02 (95%CI 0.93-1.10)
- 9-10 hours: model 1 aRR 0.89 (95%CI 0.79-1.01); model 2 aRR 0.94 (95%CI 0.84-1.06)
- >10 hours: model 1 aRR 0.82 (95%CI 0.69-0.99); model 2 aRR 0.86 (95%CI 0.72-1.03)

Self-reported bed time before pregnancy was not associated with gross motor skills (21:00-24:00 reference group):

- 24:00-03:00: model 1 aRR 1.01 (95%CI 0.95-1.09); model 2 aRR 0.97 (95%CI 0.90-1.05)
- Other: model 1 aRR 1.01 (95%CI 0.83-1.24); model 2 aRR 1.06 (95%CI 0.86-1.30)

Self-reported sleep duration before pregnancy was not associated with fine motor skills (7-8 hours reference group):

- <6 hours: model 1 aRR 1.01 (95%CI 0.88-1.15); model 2 aRR 1.02 (95%CI 0.90-1.17)
- 6-7 hours: model 1 aRR 1.04 (95%CI 0.95-1.13); model 2 aRR 1.05 (95%CI 0.97-1.15)
- 8-9 hours: model 1 aRR 1.01 (95%CI 0.93-1.09); model 2 aRR 0.99 (95%CI 0.91-1.08)
- 9-10 hours: model 1 aRR 1.00 (95%CI 0.90-1.12); model 2 aRR 0.98 (95%CI 0.87-1.09)
- >10 hours: model 1 aRR 0.95 (95%CI 0.80-1.12); model 2 aRR 0.93 (95%CI 0.79-1.10)

Self-reported bed time before pregnancy was not associated with fine motor skills (21:00-24:00 reference group):

- 24:00-03:00: model 1 aRR 0.96 (95%CI 0.90-1.03); model 2 aRR 1.00 (95%CI 0.93-1.07)
- Other: model 1 aRR 0.92 (95%CI 0.74-1.13); model 2 aRR 0.94 (95%CI 0.76-1.16)

Self-reported sleep duration before pregnancy was not associated with problems solving (7-8 hours reference group):

- <6 hours: model 1 aRR 1.09 (95%CI 0.95-1.25); model 2 aRR 1.06 (95%CI 0.93-1.21)
- 6-7 hours: model 1 aRR 1.08 (95%CI 0.98-1.18); model 2 aRR 1.04 (95%CI 0.95-1.14)
- 8-9 hours: model 1 aRR 1.00 (95%CI 0.92-1.09); model 2 aRR 1.05 (95%CI 0.96-1.15)
- 9-10 hours: model 1 aRR 1.03 (95%CI 0.91-1.16); model 2 aRR 1.12 (95%CI 1.00-1.26)
- >10 hours: model 1 aRR 1.04 (95%CI 0.88-1.24); model 2 aRR 1.10 (95%CI 0.93-1.31)

Self-reported bed time before pregnancy was not associated with problems solving (21:00-24:00 reference group):

- 24:00-03:00: model 1 aRR 1.08 (95%CI 1.00-1.16); model 2 aRR 1.00 (95%CI 0.93-1.08)
- Other: model 1 aRR 0.89 (95%CI 0.71-1.12); model 2 aRR 0.90 (95%CI 0.72-1.13)

Self-reported sleep duration before pregnancy was not associated with personal-social characteristics (7-8 hours reference group):

- <6 hours: model 1 aRR 1.11 (95%CI 0.82-1.51); model 2 aRR 1.20 (95%CI 0.88-1.62)

	▪ 6-7 hours: model 1 aRR 1.05 (95%CI 0.85-1.29); model 2 aRR 1.12 (95%CI 0.91-1.37)<br>▪ 8-9 hours: model 1 aRR 1.33 (95%CI 1.11-1.60); model 2 aRR 1.23 (95%CI 1.02-1.48)<br>▪ 9-10 hours: model 1 aRR 1.48 (95%CI 1.17-1.87); model 2 aRR 1.30 (95%CI 1.03-1.65)<br>▪ >10 hours: model 1 aRR 1.27 (95%CI 0.89-1.82); model 2 aRR 1.28 (95%CI 0.82-1.69)<br>Self-reported bed time before pregnancy was not associated with personal-social characteristics (21:00-24:00 reference group):<br>▪ 24:00-03:00: model 1 aRR 0.91 (95%CI 0.78-1.07); model 2 aRR 1.18 (95%CI 0.93-1.29)<br>▪ Other: model 1 aRR 0.66 (95%CI 0.38-1.14); model 2 aRR 0.72 (95%CI 0.41-1.24)<br><br>Self-reported sleep duration before pregnancy was not associated with total (abnormal score for any 1 of the 5 domain) (7-8 hours reference group):<br>▪ <6 hours: model 1 aRR 1.01 (95%CI 0.94-1.10); model 2 aRR 1.01 (95%CI 0.93-1.09)<br>▪ 6-7 hours: model 1 aRR 1.04 (95%CI 0.99-1.10); model 2 aRR 1.03 (95%CI 0.98-1.08)<br>▪ 8-9 hours: model 1 aRR 0.98 (95%CI 0.93-1.03); model 2 aRR 0.99 (95%CI 0.95-1.05)<br>▪ 9-10 hours: model 1 aRR 0.96 (95%CI 0.89-1.03); model 2 aRR 0.99 (95%CI 0.92-1.06)<br>▪ >10 hours: model 1 aRR 1.00 (95%CI 0.90-1.10); model 2 aRR 1.02 (95%CI 0.92-1.13)<br>Self-reported bed time before pregnancy was not associated with total (abnormal score for any 1 of the 5 domain) (21:00-24:00 reference group):<br>▪ 24:00-03:00: model 1 aRR 1.01 (95%CI 0.97-1.05); model 2 aRR 0.98 (95%CI 0.94-1.03)<br>▪ Other: model 1 aRR 0.94 (95%CI 0.82-1.07); model 2 aRR 0.97 (95%CI 0.85-1.10)
Okada *et al*. (2019)	Increase in morning systolic blood pressure from first to third trimester was larger in the good sleep quality group versus the poor sleep quality group (3.0 ±5.6 mmHg versus 7.1 ±7.0 mmHg, p<.01). Same was found for evening systolic blood pressure (3.2 ±5.7 mmHg and 6.6 ±5.9 mmHg, p=.00).<br><br>Change in morning systolic blood pressure from the first to the third trimester was influenced by the PSQI total score (r=0.49, β=0.58, p=.00). Sleep latency (r=0.38, β=0.43, p=.02) and sleep disturbances (r=0.24, β=0.33, p=.04) were the two most important components affecting this change. Other PSQI subscales did not reach statistical significance: subjective sleep quality (r=0.33; β=0.30; p=.06), sleep duration (r=0.26; β=0.36; p=.06), sleep efficiency (r=0.37; β=0.15; p=.39).
Okun *et al.* (2007)	Poorer subjective sleep quality from the PSQI in the first trimester was related to higher TNF-α levels (ρ=0.41, p=.02). Associations with all other biomarkers were non-significant (IL-4, IL-6, IL-10, CRP).
Okun *et al.* (2013)[b]	Based on sleep diaries:<br>▪ NuPDQ scores (assessing pregnancy distress) did not differ between sleep deficiency groups at 10-12 weeks.<br>▪ IDS scores (assessing depressive symptoms) differed by sleep deficiency group at 10-12 weeks ($F_{2,157}$=4.27; p=.01), with those having sleep deficiency having higher IDS scores than those with no sleep deficiency, indicating more depressive symptoms. After adjustment, this association was no longer found.

	▪ PSS scores (assessing stress) differed by sleep deficiency group at 10-12 weeks ($F_{2,157}$=3.51; p=.03), with those having sleep deficiency having higher PSS scores than those with no sleep deficiency, indicating more perceived stress. After adjustment, this association was no longer found.<br><br>Based on actigraphy:<br>▪ IDS scores (assessing depressive symptoms) did not differ between sleep deficiency groups at 10-12 weeks.<br>▪ At 10-12 weeks, NuPDQ scores (assessing pregnancy distress) differed by sleep deficiency group ($F_{2,157}$=3.96; p=.02), with those with persistent sleep deficiency having significantly higher NuPDQ scores than those with no sleep deficiency (p<.01) or intermittent sleep deficiency (p=.02), indicating more pregnancy distress.<br>▪ At 10-12 weeks PSS scores differed by sleep deficiency group ($F_{2,157}$=6.36; p<.01). Those with persistent sleep deficiency had higher PSS scores than those with no sleep deficiency (p<.01) or intermittent sleep deficiency (p<.01-.04), indicating more perceived stress. Following adjustment, differences in PSS scores among sleep deficiency groups found remained at 1-12 weeks ($F_{2,152}$=4.57, p=.01).
Rawal *et al*. (2017)	First-trimester sleep duration was not associated with subsequent gestational diabetes risk, raw or when covariates are adjusted for. Also not when studying nonobese and obese women separately.<br><br>All women (8-9 hours of sleep as reference group):<br>▪ 5-6 hours: crude RR 0.88 (95%CI 0.51-1.52); model 1 aRR 0.87 (95%CI 0.49-1.55); model 2 aRR 0.87 (0.49-1.54)<br>▪ 7 hours: crude RR 0.90 (95%CI 0.51-1.57); model 1 aRR 0.91 (95%CI 0.51-1.60); model 2 aRR 0.90 (95%CI 0.51-1.60)<br>▪ ≥10 hours: crude RR 0.90 (95%CI 0.56-1.44); model 1 aRR 1.07 (95%CI 0.67-1.71); model 2 aRR 1.04 (95%CI 0.65-1.68)<br><br>Nonobese women (8-9 hours of sleep as reference group):<br>▪ 5-6 hours: crude RR 1.01 (95%CI 0.52-1.97); model 1 aRR 1.06 (95%CI 0.53-2.13); model 2 aRR 1.06 (95%CI 0.53-2.12)<br>▪ 7 hours: crude RR 1.08 (95%CI 0.57-2.07); model 1 aRR 1.02 (95%CI 0.52-1.98); model 2 aRR 1.02 (95%CI 0.52-1.98)<br>▪ ≥10 hours: crude RR 0.92 (95%CI 0.51-1.66); model 1 aRR 1.17 (95%CI 0.64-2.14); model 2 aRR 1.09 (95%CI 0.59-2.02)<br><br>Obese women (8-9 hours of sleep as reference group):<br>▪ 5-6 hours: crude RR 0.56 (95%CI 0.21-1.45); model 1 aRR 0.60 (95%CI 0.22-1.62); model 2 aRR 0.58 (95%CI 0.22-1.56)<br>▪ 7 hours: crude RR 0.58 (95%CI 0.18-1.88); model 1 aRR 0.70 (95%CI 0.22-2.19); model 2 aRR 0.69 (95%CI 0.22-2.15)<br>▪ ≥10 hours: crude RR 0.77 (95%CI 0.36-1.65); model 1 aRR 1.00 (95%CI 0.48-2.05); model 2 aRR 1.07 (95%CI 0.50-2.29)

Sarberg *et al.* (2014)	There were no differences between snorer groups regarding systolic blood pressure (p=.779) and diastolic blood pressure (p=.053) in the first trimester.<br><br>Post hoc analyses showed that the “gestational snorers” had a significantly higher mean Epworth Sleepiness Score than the “non snorers” in the first trimester (p=.009), but this did not apply for the “habitual snorers” (p=.264).<br><br>The prevalence of excessive daytime sleepiness (EDS) defined as Epworth Sleepiness score ≥10, only differed between the groups in the first trimester of pregnancy, with significant difference between the “gestational snorers” and the “non snorers” (p=.001).<br><br>Prevalence of restless legs syndrome (RLS) in the first trimester did not differ between the groups (p=.147).
Shi *et al*. (2020)	In females, an U-shaped association was found between self-reported sleep duration and conception probability. However, not all associations were statistically significant.<br><br>USA data (7 hours of sleep as reference group):<br>▪ ≤5 hours: model 1 aRR 3.25 (95%CI 2.33-4.53); model 2 aRR 3.49 (95%CI 2.48-4.91); model 3 aRR 3.24 (95%CI 2.30-4.58)<br>▪ 6 hours: model 1 aRR 2.04 (95%CI 1.51-2.75); model 2 aRR 2.17 (95%CI 1.60-2.95); model 3 aRR 2.11 (95%CI 1.55-2.86)<br>▪ 8 hours: model 1 aRR 1.32 (95%CI 0.97-1.79); model 2 aRR 1.15 (95%CI 0.85-1.57); model 3 aRR 1.12 (95%CI 0.82-1.53)<br>▪ ≥9 hours: model 1 aRR 1.32 (95%CI 0.83-2.09): model 2 aRR 0.97 (95%CI 0.60-1.56); model 3 aRR 0.93 (95%CI 0.58-1.49)<br><br>China data (7 hours of sleep as reference group):<br>▪ ≤6 hours: model 1 aRR 1.10 (95%CI 0.57-2.11); model 2 aRR 1.30 (95%CI 0.64-2.64); model 3 aRR 1.21 (95%CI 0.58-2.54)<br>▪ 8 hours: model 1 aRR 1.38 (95%CI 0.95-2.02); model 2 aRR 1.15 (95%CI 0.77-1.74); model 3 aRR 1.12 (95%CI 0.73-1.70)<br>▪ 9 hours: model 1 aRR 1.87 (95%CI 1.21-2.90); model 2 aRR 1.31 (95%CI 0.81-2.11); model 3 aRR 1.32 (95%CI 0.81-2.17)<br>▪ ≥10 hours: model 1 aRR 2.49 (95%CI 1.60-3.88); model 2 aRR 1.57 (95%CI 0.96-2.57); model 3 aRR 1.63 (95%CI 0.97-2.75)<br><br>Each hour/day difference (longer or shorter) from 7 h/day sleep was associated with 1.26 (95%CI 1.12- 1.42, p<.001) and 1.21 (95%CI 1.03-1.41, p=.019) fold conception probability in the populations from the USA and China, respectively.

Stocker *et al.* (2020)	No statistical significant differences in PSQI or ESS self-report measures between the groups.<br>▪ PSQI subscale sleep duration (p=.48 for RM versus comparison; p=.21 for RIF versus comparison)<br>▪ PSQI subscale sleep disturbance (p=.38 for RM versus comparison; p=.35 for RIF versus comparison)<br>▪ PSQI subscale sleep latency (p=.69 for RM versus comparison; p=.67 for RIF versus comparison)<br>▪ PSQI subscale daytime dysfunction (p=.79 for RM versus comparison; p=.50 for RIF versus comparison)<br>▪ PSQI subscale sleep efficiency (p=.82 for RM versus comparison; p=.09 for RIF versus comparison)<br>▪ PSQI subscale sleep quality (p=.73 for RM versus comparison; p=.49 for RIF versus comparison)<br>▪ PSQI total score (p=.69 for RM versus comparison; p=.38 for RIF versus comparison)<br>▪ ESS total score (p=.34 for RM versus comparison; p=.42 for RIF versus comparison)<br><br>No statistical significant differences in sleep diary measures between the groups.<br>▪ Awakening time (p=.61 for RM versus comparison; p=.75 for RIF versus comparison)<br>▪ Out of bed time (p=.36 for RM versus comparison; p=.53 for RIF versus comparison)<br>▪ Bed time (p>.99 for RM versus comparison; p=.29 for RIF versus comparison)<br>▪ Time fell asleep (p=.98 for RM versus comparison; p=.35 for RIF versus comparison)<br>▪ Timing of naps (p=.71 for RM versus comparison; p=.26 for RIF versus comparison)<br>▪ Duration of naps (p=.69 for RM versus comparison; p=.86 for RIF versus comparison)<br>▪ Sleep time (p=.86 for RM versus comparison; p=.27 for RIF versus comparison)<br>▪ Morning latency (p=.61 for RM versus comparison; p=.77 for RIF versus comparison<br>▪ Night time latency (p=.42 for RM versus comparison; p=.56 for RIF versus comparison)<br><br>For the actigraphy parameters, women with RIF slept 52.72 minutes more than the control group (p=.03). All other actigraph differences were insignificant:<br>Sleep:<br>▪ Time in bed (p=.19 for RM versus comparison; p=.24 for RIF versus comparison)<br>▪ Time to sleep onset (p=.32 for RM versus comparison; p=.17 for RIF versus comparison)<br>▪ Sleep duration (p=.13 for RM versus comparison)<br>▪ Wake after sleep onset (p=.32 for RM versus comparison; p=.51 for RIF versus comparison)<br>▪ Total activity count (p=.16 for RM versus comparison; p=.61 for RIF versus comparison)<br>▪ Average activity (p=.44 for RM versus comparison; p=1.00 for RIF versus comparison)<br>▪ Efficiency (p=.18 for RM versus comparison; p=.80 for RIF versus comparison)<br>▪ Wake time (p=.25 for RM versus comparison; p=.35 for RIF versus comparison)<br>▪ Percentage awake (p=.62 for RM versus comparison; p=.67 for RIF versus comparison)<br>▪ Percentage sleep (p=.62 for RM versus comparison; p=.67 for RIF versus comparison)<br>Wake:<br>▪ Activity duration (p=.62 for RM versus comparison; p=.92 for RIF versus comparison)<br>▪ Total activity count (p=.80 for RM versus comparison; p=.49 for RIF versus comparison)<br>▪ Activity count/minute (p=.96 for RM versus comparison; p=.84 for RIF versus comparison)<br>▪ Sleep time (p=.46 for RM versus comparison; p=.59 for RIF versus comparison)<br>▪ Percentage sleep (p=.93 for RM versus comparison; p=.79 for RIF versus comparison)

	Rest:<br>▪ Rest duration (p=.12 for RM versus comparison; p=.12 for RIF versus comparison)<br>▪ Total activity count (p=.14 for RM versus comparison; p=.49 for RIF versus comparison)<br>▪ Activity count/minute (p=.39 for RM versus comparison; p=.82 for RIF versus comparison)
Tsai *et al*. (2016)	Cross-sectionally, more daytime sleep (β=0.03; p=.04) and higher PSQI total score (β=-1.07; p<.01) in the first trimester were associated with better physical health-related quality of life. Sleep efficiency (β=-0.01; p=.93), wake after sleep onset (β=0.01; p=.85), and total nighttime sleep (β=-0.81; p=.30) in the first trimester were not associated with better physical health-related quality of life.<br>Cross-sectionally, higher PSQI total score (β=-1.40; p<.01) in the first trimester was associated with better mental health-related quality of life. Total daytime sleep (β=0.02; p=.46), sleep efficiency (β=-0.01; p=.95), wake after sleep onset (β=0.01; p=.83), and total nighttime sleep (β=0.46; p=.64) in the first trimester were not associated with better mental health-related quality of sleep.<br><br>Longitudinally, total nighttime sleep (β=1.38; p=.01) and higher total PSQI score (β=-0.85; p<.01) in the first trimester predicted better mental health-related quality of life in the second trimester. Total daytime sleep (β=-0.01; p=.36), sleep efficiency (β=-0.09; p=.52), and wake after onset (β=-0.05; p=.12) in the first trimester did not predict better mental health-related quality of life in the second trimester.<br>Longitudinally, higher total PSQI score (β=-0.87; p<.01) in the first trimester predicted better physical health-related quality of life in the second trimester. Total daytime sleep (β=0.01; p=.55), sleep efficiency (β=-0.10; p=.47), wake after sleep onset (β=-0.02; p=.56), and total nighttime sleep (β=-0.12; p=.87) in the first trimester did not predict better mental health-related quality of life in the second trimester.<br><br>Longitudinally, longer total nighttime sleep (β=1.53; p=.02) and higher total PSQI score (β=-1.00; p<.01) in the first trimester predicted better mental health-related quality of life in the third trimester. Total daytime sleep (β=0.01; p=.43), sleep efficiency (β=0.07; p=.62), and wake after onset (β=-0.03; p=.39) in the first trimester did not predict better mental health-related quality of life in the third trimester.<br>Longitudinally, wake after sleep onset (β=-0.07; p=.05) predicted better physical health-related quality of life. Total PSQI score (β=-0.20; p=.41), total daytime sleep (β=-0.01; p=.59), sleep efficiency (β=-0.27; p=.06), and total nighttime sleep (β=-0.13; p=.86) in the first trimester did not predict better physical health-related quality of life.
Willis *et al.* (2019)	Shorter self-reported sleep duration (<6 hours) was associated with lower fecundibility, but not after adjustment for confounders.<br><br>Associations between sleep duration and fecundibility rate (8 hours of sleep as reference group):<br>▪ <6 hours: crude FR 0.60 (95%CI 0.67-0.94); aFR 0.89 (95%CI 0.75-1.06)<br>▪ 6 hours: crude FR 0.89 (95%CI 0.81-0.98); aFR 0.95 (95%CI 0.86-1.04)<br>▪ 7 hours: crude FR 0.98 (95%CI 0.91-1.05); aFR 0.99 (95%CI 0.92-1.06)<br>▪ ≥9 hours: crude FR 0.91 (95%CI 0.80-1.03); aFR 0.96 (95%CI 0.84-1.10)<br>More self-reported sleeping problems were associated with lower fecundibility rate (no problems as reference group):

	▪ For <50% of the time: crude FR 0.91 (95%CI 0.86-0.97); aFR 0.93 (95%CI 0.88-1.00).<br>▪ For >50% of the time: crude FR 0.80 (95%CI 0.73-0.87); aFR 0.87 (95%CI 0.79-0.95).<br><br>Restricted to women with partner data available, this did not change the findings (8 hours of sleep as reference group):<br>▪ <6 hours: crude FR 0.78 (95%CI 0.56-1.08); model 1 aFR 1.00 (95%CI 0.71-1.41); model 2 aFR 1.01 (95%CI 0.71-1.42)<br>▪ 6 hours: crude FR 0.80 (95%CI 0.68-0.95); model 1 aFR 0.85 (95%CI 0.72-1.02); model 2 aFR 0.86 (95%CI 0.71-1.03)<br>▪ 7 hours: crude FR 0.95 (95%CI 0.84-1.08); model 1 aFR 0.94 (95%CI 0.83-1.07); model 2 aFR 0.94 (95%CI 0.83-1.07)<br>▪ ≥9 hours: crude FR 0.82 (95%CI 0.65-0.98); model 1 aFR 0.86 (95%CI 0.67-1.09); model 2 aFR 0.86 (95%CI 0.67-1.09)<br>Similar to self-reported sleeping problems. Restricted to women with partner available data, more self-reported sleeping problems remained associated with lower fecundibility rate (no problems as reference group):<br>▪ For <50% of the time: crude FR 0.89 (95%CI 0.79-1.00); model 1 aFR 0.92 (95%CI 0.82-1.04); model 2 aFR 0.93 (95%CI 0.82-1.04).<br>▪ For >50 of the time: crude FR 0.76 (95%CI 0.64-0.90); model 1 aFR 0.83 (95%CI 0.69-0.99); model 2 aFR 0.83 (95%CI 0.70-1.00).
Yu *et al.* (2017)	Cross-sectionally, sleep duration in the first trimester was associated with depressive symptoms (β=-0.28; SE=0.08; p<.01) and anxiety symptoms (β=-0.33; SE=0.07; p<.01).<br><br>In cross-sectional analysis, women who slept <8 hours per day had higher risk of depression (OR 1.75; 95%CI 1.39-2.20) and anxiety (OR 2.00, 95%CI 1.57-2.55) in the first trimester.<br><br>Also, women who reported to have a fair a bad sleep quality had a higher risk of depression or anxiety in the first trimester.<br>▪ Fair: OR 1.57 (95%CI 1.34-1.84) for depression and OR 2.52 (95%CI 2.06-3.09) for anxiety.<br>▪ Bad: OR 3.27 (95%CI 2.28-4.32) for depression and OR 7.39 (95%CI 5.89-10.67) for anxiety.
Yun *et al.* (2021)	Self-reported insufficient sleep before pregnancy and at 12 weeks were statistically significantly associated with postpartum depression at 4 weeks (respectively, OR 1.37 (95%CI 1.07-1.75); p=0.013, and OR 1.43 (95%CI 1.11-1.83); p=0.005). This was not the case after adjusting for covariates.

[a] Facco *et al*. (2018) and Facco *et al.* (2019) analysed the same study population; b Haney *et al*. (2014) and Okun *et al*. (2013) studied the same study population; [c] Nakahara (2020) and (2021) studied the same population.
AIS = Athens Insomnia Scale; BMI = body mass index; ESS = Epworth Sleepiness Scale; PSQI = Pittsburgh Sleep Quality Index.

Figure S1. Risk of bias assessment per study, based on the ErasmusAGE (N=27).

**Studies**	**Study design**	**Study size**	**Exposure assessment**	**Outcome assessment**	**Adjustment**
Bublitz *et al.* (2010)	Yellow	Yellow	Green	Green	Green
Chang *et al.* (2015)	Red	Red	Green	Green	Red
Doyon *et al.* (2020)	Yellow	Green	Yellow	Yellow	Yellow
Facco *et al.* (2018)	Yellow	Green	Yellow	Green	Green
Facco *et al.* (2019)	Yellow	Green	Yellow	Green	Green
Franco-Sena *et al*. (2018)	Yellow	Yellow	Yellow	Green	Green
Gelaye *et al*. (2015)	Red	Green	Green	Green	Green
Georgiou *et al*. (2019)	Yellow	Red	Red	Green	Red
Haney *et al.* (2014)	Yellow	Yellow	Green	Green	Yellow
Hill *et al.* (2020)	Yellow	Yellow	Green	Green	Red
Liu *et al.* (2019)	Yellow	Green	Green	Green	Green
Lyu *et al*. (2020)	Yellow	Green	Red	Green	Green
Marinelli *et al.* (2021)	Yellow	Green	Yellow	Green	Green
Matsuo *et al.* (2021)	Yellow	Green	Yellow	Green	Green
Nakahara *et al.* (2020)	Yellow	Green	Yellow	Yellow	Green
Nakahara et al. (2021)	Yellow	Green	Yellow	Green	Green
Okada *et al*. (2019)	Yellow	Red	Green	Yellow	Yellow
Okun *et al.* (2007)	Yellow	Red	Green	Green	Yellow
Okun *et al.* (2013)	Yellow	Yellow	Green	Green	Yellow
Rawal *et al*. (2017)	Yellow	Green	Yellow	Green	Green
Sarberg *et al.* (2014)	Yellow	Yellow	Yellow	Green	Red
Shi *et al*. (2020)	Yellow	Green	Yellow	Yellow	Green
Stocker *et al.* (2020)	Yellow	Red	Green	Green	Red
Tsai *et al*. (2016)	Yellow	Yellow	Green	Green	Yellow
Willis *et al.* (2019)	Yellow	Green	Yellow	Green	Green
Yu *et al.* (2017)	Yellow	Green	Yellow	Green	Yellow
Yun *et al.* (2021)	Yellow	Green	Yellow	Green	Yellow

Green = low risk of bias; yellow = unclear/moderate risk of bias; red = high risk of bias.

Figure S2. Risk of bias assessment per criterion, based on the ErasmusAGE (N=27).

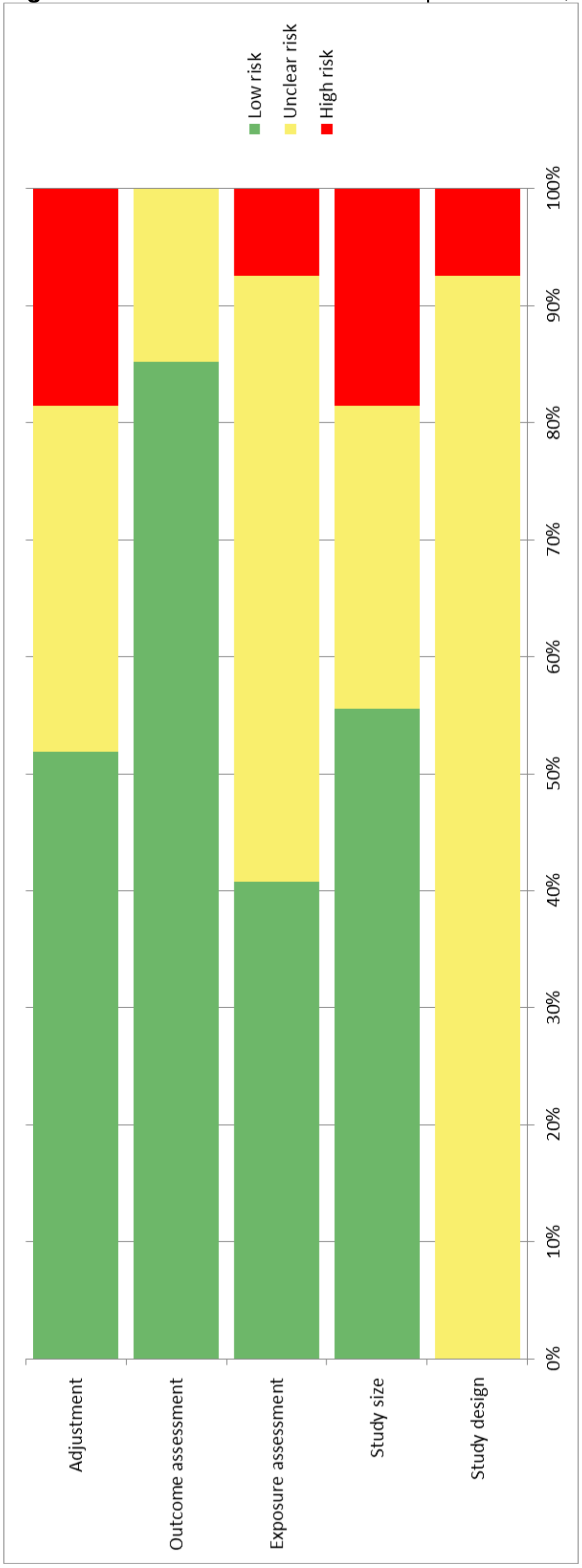

This figure shows a summary of the risk of bias assessment per article, shown in Figure S1. Green = low risk of bias; yellow = unclear/moderate risk of bias; red = high risk of bias.
